# Genome Diversity and Divergence in *Drosophila mauritiana*: Multiple Signatures of Faster X Evolution

**DOI:** 10.1093/gbe/evu198

**Published:** 2014-09-09

**Authors:** Daniel Garrigan, Sarah B. Kingan, Anthony J. Geneva, Jeffrey P. Vedanayagam, Daven C. Presgraves

**Affiliations:** Department of Biology, University of Rochester

**Keywords:** adaptation, *Drosophila*, genome, satellite DNA, selective sweep, X chromosome

## Abstract

*Drosophila mauritiana* is an Indian Ocean island endemic species that diverged from its two sister species, *Drosophila simulans* and *Drosophila sechellia*, approximately 240,000 years ago. Multiple forms of incomplete reproductive isolation have evolved among these species, including sexual, gametic, ecological, and intrinsic postzygotic barriers, with crosses among all three species conforming to Haldane’s rule: F_1_ hybrid males are sterile and F_1_ hybrid females are fertile. Extensive genetic resources and the fertility of hybrid females have made *D. mauritiana*, in particular, an important model for speciation genetics. Analyses between *D. mauritiana* and both of its siblings have shown that the X chromosome makes a disproportionate contribution to hybrid male sterility. But why the X plays a special role in the evolution of hybrid sterility in these, and other, species remains an unsolved problem. To complement functional genetic analyses, we have investigated the population genomics of *D. mauritiana*, giving special attention to differences between the X and the autosomes. We present a de novo genome assembly of *D. mauritiana* annotated with RNAseq data and a whole-genome analysis of polymorphism and divergence from ten individuals. Our analyses show that, relative to the autosomes, the X chromosome has reduced nucleotide diversity but elevated nucleotide divergence; an excess of recurrent adaptive evolution at its protein-coding genes; an excess of recent, strong selective sweeps; and a large excess of satellite DNA. Interestingly, one of two centimorgan-scale selective sweeps on the *D. mauritiana* X chromosome spans a region containing two *sex-ratio* meiotic drive elements and a high concentration of satellite DNA. Furthermore, genes with roles in reproduction and chromosome biology are enriched among genes that have histories of recurrent adaptive protein evolution. Together, these genome-wide analyses suggest that genetic conflict and frequent positive natural selection on the X chromosome have shaped the molecular evolutionary history of *D. mauritiana*, refining our understanding of the possible causes of the large X-effect in speciation.

## Introduction

During the past 30 years, the three species of the *Drosophila simulans* clade—*D. simulans, Drosophila sechellia*, and *Drosophila mauritiana*—have emerged as important models in evolutionary genetics, owing mostly to their phylogenetic proximity to *Drosophila melanogaster*. The common ancestor of the *D. simulans* clade species originated on Madagascar approximately 3 Ma, splitting from that of *D. melanogaster*, and later gave rise to *D. sechellia* on the Seychelles archipelago and to *D. mauritiana* on Mauritius nearly simultaneously, approximately 240 ka (Lachaise et al. 1986; Ballard 2000a; Kliman et al. 2000; Dean and Ballard 2004; Kopp et al. 2006; McDermott and Kliman 2008; Garrigan et al. 2012). *Drosophila mauritiana* is an island endemic species, and *D. simulans*, despite having a recently established cosmopolitan distribution, has never been collected on Mauritius (David et al. 1989; Legrand et al. 2011). Nevertheless, although *D. mauritiana* and *D. simulans* have had largely allopatric histories, there is evidence for limited interisland migration and natural hybridization. Multiple mitochondrial haplotypes have introgressed from *D. simulans* into *D. mauritiana* (Ballard 2000b; Nunes et al. 2010), and evidence for interspecific introgression, probably from *D. simulans* into *D. mauritiana*, is scattered over all three major chromosomes (Garrigan et al. 2012). However, genomic introgressions between the *D. simulans* and *D. mauritiana* are underrepresented on the X chromosome (Garrigan et al. 2012), suggesting that X-linked chromosomal segments are less exchangeable between species than those on the autosomes.

The paucity of interspecific introgressions on the X chromosome may be attributable to selection against hybrid incompatibilities that cause intrinsic postzygotic isolation. Crosses between *D. mauritiana* and its two sister species follow Haldane’s rule: all crosses yield sterile F_1_ hybrid (XY) males but fertile F_1_ hybrid (XX) females. Genetic analyses involving *D. mauritiana* show that the X chromosome has a disproportionately large effect on hybrid male sterility (HMS; Coyne 1985, 1992; Coyne and Charlesworth 1989). This so-called large X-effect has two proximate causes: recessive HMS factors on the X chromosome are fully expressed in hemizygous hybrid males (Turelli and Orr 1995, 2000); and between *D. mauritiana* and its siblings species, the density of HMS factors on the X chromosome is 2–4 times greater than on the autosomes (True, Weir, et al. 1996; Tao et al. 2003; Masly and Presgraves 2007). The large X-effect holds across a range of taxa, including other *Drosophila* (Moehring et al. 2006), mammals (Good et al. 2008), fish (Kitano et al. 2009), butterflies (Naisbit et al. 2002), and birds (Saetre et al. 2001). Determining the evolutionary cause(s) of the large X-effect is thus one of the major unsolved problems of speciation genetics (Charlesworth et al. 1987; Coyne and Orr 1989; Coyne 1992; Presgraves 2008b). There are three leading candidate explanations. First, X–autosome differences in transcriptional regulation exist in the male germline that may be especially prone to disruption by hybrid incompatibilities (Lifschytz and Lindsley 1971; Wu and Davis 1993; Hense et al. 2007; Meiklejohn et al. 2011). Second, the X chromosome is susceptible to the invasion and spread of selfish genetic elements that may, as incidental byproducts, contribute to sterility in species hybrids (Frank 1991; Hurst and Pomiankowski 1991; Tao et al. 2001; Presgraves 2008a, 2008b). Third, the X chromosome may experience a faster rate of adaptive evolution than the autosomes which could, in turn, contribute to the evolution of more X-linked hybrid incompatibilities (Charlesworth et al. 1987; Coyne and Orr 1989; Presgraves 2008b; Hvilsom et al. 2012; Llopart 2012; Meisel et al. 2012; Meisel and Connallon 2013; Kousathanas et al. 2014). These explanations are not mutually exclusive and, while evidence for each exists (Phadnis and Orr 2009; Campbell et al. 2013), their relative importance is still unknown.

To complement functional genetic analyses of speciation and the large X-effect, we have performed population genomics analyses of polymorphism and divergence for ten genomes sampled from *D. mauritiana*. Our analyses show that the *D. mauritiana* X chromosome bears more signatures of recent, strong, and recurrent positive natural selection than the autosomes. Genes and gene regions with signatures of positive selection are enriched for functions in gametogenesis, chromosome biology, and satellite DNA, implying that genetic conflict over transmission contributes to molecular evolution in *D. mauritiana*.

## Materials and Methods

### Fly Inbred Lines, Library Preparation, and Sequencing

We sampled nine wild-type isofemale lines of *D. mauritiana* (collected in 2006 and kindly donated by Maria Ramos–Womack) and the inbred laboratory strain, *mau12* (14021-0241.60). Wild-type lines were subjected to single-pair sibling mating for a minimum of nine generations. Genomic DNA extraction and library preparation were performed for pooled females as previously described (Garrigan et al. 2012). However, additional sequences for the *mau12* line were collected from a large-insert, paired-end Nextera library (∼3,000 bp) and a TrueSeq paired-end RNA library (∼260 bp). For the large-insert library, we pooled females and purified genomic DNA from whole flies using the DNeasy Blood and Tissue kit (Qiagen, Valencia, CA). DNA concentration was determined with the Qubit Flourometer (Life Technologies, Grand Island, NY) and quality was assessed using the Agilent Bioanalyzer (Agilent, Santa Clara, CA). Mate pair libraries were generated using the Nextera Mate Pair protocol (Illumina, San Diego, CA). Briefly, 4 μg of genomic DNA was fragmented per manufacturer’s protocol followed by strand displacement, agarose gel size selection of 5-kb target size, and circularization. The 5-kb fragments were sheared using a Covaris S2 (Covaris Inc., Woburn, MA) to 300- to 1,000-bp size fragments for end repair, A-tailing, and indexed adaptor ligation. The amplified libraries were purified by AMPure (Beckman Coulter, Brea, CA) purification and hybridized to an Illumina paired-end flow cell for cluster amplification using the cBot (Illumina) at a concentration of 8 pmol per lane.

For RNAseq, males and females were reared on standard cornmeal food at room temperature. 50% of males and 50% of females were allowed to freely mate for 3 days, the rest were maintained as virgins. Adults were aged 4–5 days then flash frozen in liquid nitrogen and stored at −80 °C. Total RNA was isolated from whole tissue using the RNeasy Plus Kit (Qiagen) per manufacturer’s recommendations. RNA concentration was determined with the NanoDrop 1000 spectrophotometer (NanoDrop, Wilmington, DE) and RNA quality was assessed with the Agilent Bioanalyzer (Agilent). The TruSeq RNA Sample Preparation Kit V2 (Illumina) was used for next generation sequencing library construction per manufacturer’s protocols. Briefly, polyA mRNA was purified from approximately 100 ng total RNA with oligo-dT magnetic beads and fragmented. First-strand cDNA synthesis was performed with random hexamer priming followed by second-strand cDNA synthesis. End repair and 3′ adenylation was performed on the double-stranded cDNA. Illumina-indexed adaptors were ligated to both ends of the cDNA, purified by gel electrophoresis, and amplified with polymerase chain reaction primers specific to the adaptor sequences to generate amplicons of approximately 200–500 bp in size. The amplified libraries were purified by AMPure (Beckman Coulter) purification and hybridized to an Illumina paired end flow cell for cluster amplification using the cBot (Illumina) at a concentration of 8 pmol per lane.

Each of the three additional *mau12* libraries (large-insert, male RNAseq, and female RNAseq) were bar-coded and multiplexed on the equivalent of one-half of a single flow cell. Paired-end (2×100 bp) sequencing was performed per manufacturer’s recommendations. The raw data were demultiplexed using configurebcl2fastq.pl version 1.8.3. Low complexity reads and vector contamination were removed using sequence cleaner (seqclean) and the National Center for Biotechnology Information (NCBI) univec database, respectively. The FASTX toolkit (fastq_quality_trimmer) was applied to remove bases with Phred quality scores below *Q* = 13 from the end of each read.

### De Novo Genome Assembly

Both the short- and long-insert libraries were used for de novo assembly of the *mau12* genome. Raw sequence reads were first filtered by discarding reads with 1) more than 25% of bases called as ambiguous, 2) more than 40% of bases masked as low sequence complexity, and 3) containing either vector or adapter sequences. The ABYSS genome assembler software was then used to assemble reads from both libraries simultaneously (Simpson et al. 2009) with a *k*-mer size of 64 bp. Statistics describing the *mau12* de novo genome assembly can be found in supplementary table S1, Supplementary Material online.

The resulting contigs were ordered first by identifying contigs containing the flanking sequence from the *P* [*lac-w*^+^]-element insertion sites (Araripe et al. 2006). A total of 69 contigs could be ordered using the *P*-element insertion sites, which represents nearly 39 Mb of sequence. Additional contigs were ordered by assuming synteny with *D. simulans*. The NUCMER program (Kurtz et al. 2004) was used to align all *mau12* contigs to the reference sequence for the *D. simulans w*^501^ strain (Hu et al. 2013). Contigs were then ordered by placing uniquely mapping contigs in the same order as the *w*^501^ genomic sequence with the highest sequence identity. There was no resulting conflict between placing contigs in this manner with the order inferred from the *P*-element insertion sites (data not shown). Finally, syntenic pseudochromosomes were constructed by inferring regions of overlap between adjacent contigs and filling gaps with IUPAC ambiguity characters, such that the total chromosome length of the *mau12* draft is the same as the *w*^501^ chromosomes (supplementary table S2, Supplementary Material online). Finally, unincorporated contigs were blasted to the NCBI database. Unincorporated contigs with bacterial or viral homology were excluded, whereas those with homology to insects or those with no known homology were retained for further analysis.

### Short Read Alignment

The BWA-MEM alignment algorithm (Li 2013) was used to map sequence reads from each of the ten lines of *D. mauritiana* and two lines of *D. simulans*, the MD063 strain from Madagascar (Garrigan et al. 2012) and the *w*^501^ strain (Hu et al. 2013). Additionally, short reads from the *D. melanogaster* reference strain *y; cn bw; sp* were aligned to the *mau12* de novo draft genome (see supplementary table S3, Supplementary Material online, for SRA accession numbers). Reads that did not properly pair in mapping or that had a Phred-scaled mapping score less than 30 were discarded. Additionally, to increase the accuracy of variant calling, all duplicate sequences were removed and reads were realigned around flanking indels using the SAMTOOLS software (Li et al. 2009). The total number of reads mapping to the *mau12* reference genome per library, after quality filtering, is provided in supplementary table S3, Supplementary Material online. Finally, the proportion of the *mau12* reference genome covered by the short read assembly and the median read depth across the major chromosome arms are given in supplementary table S4, Supplementary Material online.

### Transcriptome Assembly and Annotation of Genome Features

To characterize the transcriptome of *mau12*, we separately mapped reads from paired-end RNA libraries of males and females to the *mau12* syntenic pseudochromosomes using BOWTIE2 (Langmead and Salzberg 2012). Transcripts were assembled using CUFFLINKS and the male and female GTF files were merged using CUFFMERGE (Trapnell et al. 2010). The largest open reading frame (ORF) was determined for each transcript using a custom Perl script, assuming canonical start and stop codons. A genome annotation file in GFF3 format (http://www.sequenceontology.org/gff3.shtml, last accessed September 12, 2014) was generated for the following fields using the GTF and ORF data for each transcript: exon, intron, coding sequences (CDS), 5′-UTR, and 3′-UTR.

### Genome Scans of Polymorphism and Divergence

Divergence of the *mau12* assembly with the reference sequences of both *D. simulans w*^501^ and *D. melanogaster* was measured as a Hamming distance in 10-kb sliding windows along whole-chromosome alignments created with the MAUVE software (Darling et al. 2010). Only windows with greater than 10% of the total number of sites being aligned were considered for analysis. Polymorphism among the ten lines of *D. mauritiana* was summarized by measuring unbiased nucleotide diversity (*π*). Estimates of *π* in each 10-kb window were corrected for unequal sample sizes by multiplying by a factor of *n*/(*n* − 1) (Nei 1987). Similarly, in each window, Tajima’s *D* statistic was calculated as a measure of the site frequency spectrum (SFS) (Tajima 1989). Because the ten lines of *D. mauritiana* were inbred for at least nine generations, we also measured linkage disequilibrium in 10-kb windows using the unweighted average pairwise *r*^2^ statistic *Z_nS_* (Kelly 1997). Singleton polymorphic sites were excluded from the calculation of *Z_nS_*. All analyses of polymorphism, the SFS, and linkage disequilibrium were performed using the POPBAM software with default quality filtering settings (Garrigan 2013). Finally, the calculation of *π*, Tajima’s *D*, and *Z_nS_* assumed haploid genotypes, thereby ignoring any residual heterozygosity that remains after inbreeding.

### Polymorphism and Divergence Analysis of Sequence Classes

We analyzed polymorphism and divergence for the following sequence classes: CDS, 5′-UTR, 3′-UTR, intron, exon, bases 8–30 of introns shorter than 70 bp (the median intron length in the *mau12* genome). We analyzed the longest transcript from each of the annotated genes, a total of 11,348 transcripts. From the BAM file described above we generated a VCF file (Danecek et al. 2011) using SAMTOOLS mpileup and BCFTOOLS view, removing reads with mapping quality less than 20 and without performing indel calling (Li et al. 2009). For estimates of *π*, we used VCFTOOLS (Danecek et al. 2011) to generate estimates of average pairwise distance using the “window-pi” function. The VCF file generated to calculate *π* contained only the ten *D. mauritiana* samples (the “-s” option in BCFTOOLS view). To calculate *π* for CDS and UTR sequences that spanned multiple fragments, we concatenated the VCF files and then renumbered the position field starting with position “1” through the total length of all concatenated fragments. To calculate all other summary statistics, we generated FASTA sequences from the VCF file using vcfutils.pl vcf2fq (part of the SAMTOOLS package) and the “seq” and “cutN” utilities of the “sequence tool kit” (SEQTK v1.0-r31, https://github.com/lh3/seqtk, last accessed September 12, 2014). For CDS and UTR sequences that spanned multiple fragments, we concatenated FASTA sequences together into a single file and reverse complemented CDS as necessary. The average Jukes–Cantor-corrected pairwise distance (Nei and Kumar 2000) between *D. mauritiana* and *D. melanogaster* was calculated from these FASTA alignments using a custom Perl script. In addition, we calculated average *d*_N_ and *d*_S_ (Yang and Nielsen 2000) and *p*_N_ and *p*_S_ using a custom Perl script calling CODEML (Yang 2007). Similar results were obtained using YN00, which is an approximate method for calculating *d*_N_ and *d*_S_ (Yang and Nielsen 2000).

McDonald–Kreitman (MK) tests were used to test the neutral model of protein evolution (McDonald and Kreitman 1991) and estimate the proportion of adaptive amino acid substitutions (Smith and Eyre-Walker 2002). Two different variants of the MK test were performed: 1) A pooled, unpolarized MK test between *D. mauritiana* and *D. melanogaster*, and 2) a lineage-specific MK test to identify *D. mauritiana*-specific mutations where *D. simulans w*^501^ was used as the near-outgroup and *D. melanogaster* as a far-outgroup. Tests were performed with custom Perl scripts using the method of Begun et al. (2007). The proportion of divergent sites driven by positive selection (α) was calculated from counts of polymorphic and fixed synonymous and nonsynonymous changes (Fay et al. 2002), after excluding singletons. A polarized and unpolarized statistic was estimated as described for MK tests. A separate measure, α*, was calculated using the short intron sequence class as our neutral standard. Again, we counted the total polymorphic sites and fixed differences, excluding singletons. Finally, 95% confidence intervals around α estimates were calculated by performing 1,000 bootstrap resampling replicates.

### Tests for Recent Selective Sweeps

Recent selective sweeps are expected to generate aberrant SFS surrounding the targets of selection. We used the parametric test described by Nielsen and colleagues, implemented in the program SweepFinder (Nielsen et al. 2005), to suggest candidate regions experiencing recent selective sweeps by identifying genomic windows with an unusual SFS. We first used POPBAM to identify all variable sites in the alignment described above. We then focused our analyses on the *D. mauritiana* lineage by filtering for sites where a derived state was present in at least one *D. mauritiana* sample, but absent in all samples from *D. melanogaster* and *D. simulans*. We calculated unfolded frequency spectra for 10-kb nonoverlapping windows and used those windows with at least 5,000 aligned sites as input for SweepFinder, which determines a maximum composite likelihood ratio for each window by contrasting the likelihood of a complete selective sweep at the location to the null hypothesis of no sweep using the observed SFS in a window and the chromosome-wide SFS. We calculated *P* values of the maximum composite likelihood ratios reported by SweepFinder using 9 degrees of freedom. Finally, contiguous tracts of windows were grouped into putative sweep regions using a simple hidden Markov model with two possible states, “sweep” and “nonsweep.” The emission probabilities are derived directly from the SweepFinder *P* values and the transition probabilities are derived from the product of the marginal frequencies of significant and nonsignificant SweepFinder windows.

### Analysis of Repetitive Sequence

The repeat content of each chromosome arm was analyzed with RepeatMasker (Smit et al. 2010). To analyze repeat-enriched regions of each chromosome, a dot plot was generated in Geneious (Kearse et al. 2012) and repeat blocks were identified by eye. Chromosome coordinates were estimated as the proportional distance along the dot plot axis. Our repeat analysis is based on the proportion of masked sequence, rather than the absolute length of masked sequence, and is robust to error in estimating these coordinates. In addition, we separately characterized the *D. simulans* 359-bp satellite, which was absent from the Repbase database used by RepeatMasker (Jurka et al. 2005). The 359-bp satellite sequences were ascertained by a BLAST search using the *D. melanogaster* canonical 359 satellite sequence (Hsieh and Brutlag 1979) against the *D. simulans* reference assembly (Hu et al. 2013); the sequence defined by coordinates X:1,218,055–1,218,405 was used as the query in the BLAST against our *mau12* reference sequence.

## Results

### De Novo Genome Assembly and Annotation

Our de novo assembly of the reference *D. mauritiana white* (*w*; here after *mau12*) has a total length of more than 124 Mb. The longest of our 2,759 scaffolds is nearly 3 Mb (supplementary table S1, Supplementary Material online). More than 50% of the assembly is contained in scaffolds ≥542 kb. We assembled chromosome-level sequences for the five major arms, using between 59 and 151 scaffolds per arm. Each chromosome-level draft sequence is ≥80% of the length of the corresponding *D. melanogaster* reference sequence. For the ten *D. mauritiana* lines, we obtained 72- to 130-fold sequence coverage spanning 98.8–99.5% of the de novo *mau12* assembly (supplementary table S4, Supplementary Material online). To annotate the de novo assembly, we generated more than 38 (28) million pairs of 100-bp RNAseq reads from the whole bodies of *mau12* females (males), after quality filtering. In total, 86% (80%) of reads from females (males) were mapped to the *mau12* pseudochromosome reference sequences using BOWTIE2. Despite the greater number of reads generated from females, CUFFLINKS predicts 15,890 transcripts (from 12,071 genes) in males, compared with 12,488 transcripts (from 9,762 genes) in females. Merging the transcript annotations of the sexes yields a total of 16,261 predicted transcripts from 11,356 genes. Note that the number of genes in the merged annotation is lower than the male annotation because overlapping or closely neighboring transcripts may be combined into a single gene model.

### Genome Diversity and Divergence

**Fig. 1.—:**
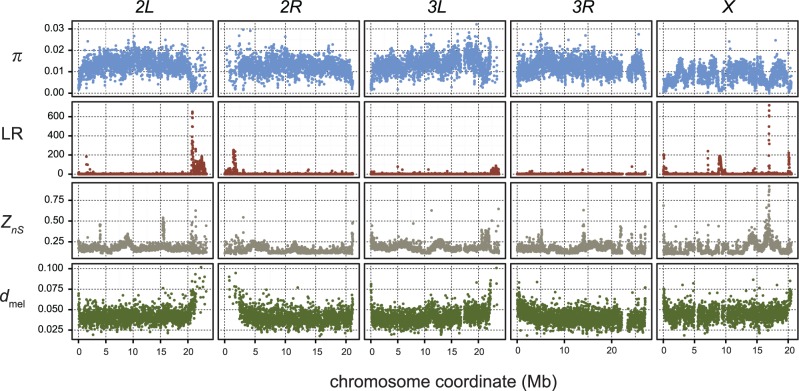
Scans of population genetics statistics across the five major chromosomal arms of the *D. mauritiana* genome. The four statistics were calculated in nonoverlapping 10-kb windows. Each column of plots represents scans from a single chromosome arm. The top row of plots (blue points) shows scans of nucleotide diversity (*π*). The second row of plots (red points) shows the distribution of a likelihood ratio test statistic that measures the deviation of the local allele frequency spectrum (LR). The third row of plots (grey points) shows the measure of linkage disequilibrium (*Z_nS_*) across all arms. Finally, the bottom row of plots (green points) shows the scan of average sequence divergence between the ten *D. mauritiana* samples and a single *D. melanogaster* genome sequence.

We surveyed nucleotide polymorphism and divergence from *D. melanogaster* in 10-kb windows for ten *D. mauritiana* genomes (fig. 1). Compared with the autosomes, the X chromosome has significantly elevated divergence (*d*_X_/*d*_A_ = 1.100), but reduced polymorphism (*π*_X_/*π*_A_ = 0.649; table 1, Mann–Whitney *U* tests, *P*_MWU_ < 2.2 × 10^−16^). These contrasting X/A ratios for polymorphism and divergence cannot be explained by a standard neutral model, assuming no selection, an equal breeding sex ratio, and constant effective population size (*N*_e_), such that *N*_e_,_X_/*N*_e,A_ = ¾. Furthermore, the observed *π*_X_/*π*_A_ ratio of nucleotide diversity is difficult to reconcile with an extreme founder event (Pool and Nielsen 2008), for which there is no evidence in the recent history of *D. mauritiana*. The X/A ratios of polymorphism and divergence are however consistent with a model involving selection. First, under a model of nearly neutral evolution, slightly deleterious substitutions can accumulate faster on the X than on the autosomes. Assuming new mutations have scaled selection coefficients of *N*_e_*s* ≈ −3 (or weaker), and assuming *N*_e,X_/*N*_e,A_ ≈ 0.65 (as observed), then the X chromosome is expected to experience a higher rate of substitution than the autosomes, regardless of dominance (Vicoso and Charlesworth 2009). Second, under a model of adaptive evolution, beneficial mutations can accumulate on the X more quickly than on the autosomes (Charlesworth et al. 1987; Vicoso and Charlesworth 2009). Assuming new beneficial mutations tend to be recessive, then the X/A ratios of polymorphism and divergence could be consistent with a model of recurrent hitchhiking in which selective sweeps on the X chromosome are more frequent, stronger, and/or more often involve new beneficial mutations rather than standing genetic variation (Begun and Whitley 2000; Orr and Betancourt 2001; Betancourt et al. 2004).

**Table 1:** Nucleotide Diversity in *Drosophila mauritiana* (*π*), Divergence between *D. mauritiana* and *Drosophila melanogaster* (*d*, Jukes–Cantor Corrected Average Pairwise Divergence), and Tajima’s *D* (*TD*) for Different Functional Classes of Site

Sequence Class	*π*_X_	*π*_A_	*π*_X_/*π*_A_	*d*_X_	*d*_A_	*d*_X_/*d*_A_	*TD*_X_	*TD*_A_	*TD*_X_/*TD*_A_
Genome	0.008	0.013	0.649*	0.045	0.041	1.100*	−0.53	−0.33	1.58*
SI8-30	0.012	0.022	0.567*	0.075	0.076	0.979*	−0.51	−0.55	0.93
Synonymous	0.011	0.015	0.757	0.109	0.102	1.065*	−0.90	−0.97	0.93*
Introns	0.007	0.011	0.653*	0.033	0.032	1.040*	−0.78	−0.81	0.96*
UTR	0.005	0.008	0.603*	0.034	0.034	1.026*	−0.95	−1.02	0.93*
Nonsynonymous	0.001	0.002	0.868*	0.014	0.011	1.260*	−0.93	−1.07	0.87*

Note.—The values given in the table are averages over all autosomal sites and all X-linked sites. The ratio of the X-linked value to that of the autosomal value is also provided. Asterisk indicates a significant departure from expectations based on selective neutrality and equal breeding sex ratio (Mann–Whitney *U* test, *P *< 0.05)

Heterogeneity in levels of polymorphism and divergence also exists within chromosome arms, in part due to local selective sweeps (see below), and in part due to the qualitatively different patterns of diversity and divergence in centromere- and telomere-proximal regions. Euchromatic regions that are centromere- and telomere-proximal have reduced rates of crossing over (True, Mercer, et al. 1996), reduced polymorphism, and elevated divergence (fig. 1; Begun et al. 2007; Langley et al. 2012; Mackay et al. 2012). These patterns imply that recurrent selective sweeps and/or background selection, and their consequent Hill–Robertson effects, impact the genomic distribution of polymorphism and divergence in the *D. mauritiana* genome, especially in regions with reduced rates of crossing over (Hill and Robertson 1966; Felsenstein 1974; Maynard-Smith and Haigh 1974; Begun and Aquadro 1992; Charlesworth et al. 1993; Hey and Kliman 2002; Langley et al. 2012; Mackay et al. 2012).

### Selective Constraint in the Genome

To characterize how natural selection has shaped sequence evolution in the *D. mauritiana* genome, we compared divergence and polymorphism across five sequence classes (table 1 and supplementary fig. S1, Supplementary Material online). Synonymous sites show the highest divergence, followed by bases 8–30 of short introns (i.e., introns shorter than the median intron length of 70 bp, hereafter SI8-30), suggesting that these are the two least constrained sequence classes. Within *D. mauritiana*, SI8–30 sites have the highest per-site estimate of *π* and the least negative Tajima’s *D*, a summary of the SFS (table 1). These findings suggest that SI8–30 sites have experienced the least selective constraint within the recent history of the species (Parsch et al. 2010). Divergence, polymorphism, and Tajima’s *D* at nonsynonymous sites are lower than those at synonymous and SI8–30 sites (table 1), consistent with strong functional constraints on protein-coding changes. Divergence, polymorphism, and Tajima’s *D* at introns and UTRs are intermediate (table 1), consistent with weaker functional constraints at these sites (Andolfatto 2005; Begun et al. 2007; Langley et al. 2012; Mackay et al. 2012).

### Different Patterns of Molecular Evolution on the X Chromosome and Autosomes

Sequence divergence between *D. mauritiana* and *D. melanogaster* is higher on the X chromosome than on the autosomes for all sequence classes (*P*_MWU_ ≤ 0.023) but one: SI8–30 site divergence on the X is significantly lower than on the autosomes (*P*_MWU_ = 0.0035; table 1). Assuming SI8–30 sites are the least constrained class, the slower X evolution observed at these sites suggests either that these positions are weakly constrained, with more effective purifying selection on the X, or that the rate of mutation is lower on the X chromosome. Tajima’s *D* at SI8–30 sites is comparable between the X and autosomes (−0.51 and −0.55, respectively, *P*_MWU_ = 0.0783), giving no indication of a chromosome-wide difference in the efficacy of purifying selection. Instead, these findings are consistent with a male germline mutation rate that is 1.14-fold higher than that in the female germline (CI_95_ = 0.91-1.42) (Miyata et al. 1987), as seen in some previous population and experimental genetic analyses in other *Drosophila* species (Bachtrog 2008; Keightley et al. 2009).

Several lines of evidence indicate more frequent positive selection on the X chromosome relative to the autosomes. First, assuming that SI8–30 sites are indeed the least constrained class (Parsch et al. 2010), then the faster X evolution observed for all other sequence classes implies more frequent positive selection at these X-linked sites. Second, for all noncoding sequence classes, polymorphism on the X chromosome is significantly reduced relative to the autosomes (*P*_MWU_ < 2 × 10^−16^), even after accounting for the chromosome difference in effective size (*N*_x_/*N*_A_ = ¾). As the SFS is comparable between X and autosomes for each sequence class (table 1; the same holds for preferred, unpreferred, and neutral synonymous polymorphisms; not shown), the reduced sequence polymorphism on the X is not readily attributable to more effective purifying selection. A strong alternative is that the reduced diversity on the X reflects more frequent hitchhiking events caused by the fixation of new beneficial mutations (Begun and Whitley 2000). Taken together, the data support a model in which the X has a slightly lower mutation rate but nevertheless experiences a higher substitution rate at most functional sequence classes owing to a greater efficacy of positive natural selection (Charlesworth et al. 1987; Kirkpatrick and Hall 2004).

### Adaptive Evolution of Protein-Coding Genes

We used MK tests (McDonald and Kreitman 1991) to evaluate the neutral mutation–drift null hypothesis for more than 11,000 protein-coding genes. We performed unpolarized MK tests, with *D. melanogaster* as the outgroup species, and polarized MK tests, to characterize lineage-specific evolution in *D. mauritiana*, with *D. melanogaster* as the far-outgroup and *D. simulans* as an additional near-outgroup. The polarized MK tests thus involve polymorphisms and fixed differences accumulated only in the *D. mauritiana* lineage. At the *P* < 0.05 level, 91 (417) genes have excess nonsynonymous substitutions for the polarized (unpolarized) MK tests, consistent with recurrent positive selection, whereas 26 (267) genes have excess nonsynonymous polymorphisms, consistent with segregating deleterious alleles or some forms of balancing selection. At the *P* < 0.01 level, 23 (144) genes have excess nonsynonymous substitutions for the polarized (unpolarized) MK tests, and 5 (89) have excess nonsynonymous polymorphisms. For the unpolarized MK tests, the X chromosome has a significant 1.6-fold excess of positively selected genes relative to autosomes at the *P* < 0.01 level using Fisher’s exact test (*P*_FET_ = 0.020), but not at the *P* < 0.05 level (*P*_FET_ = 0.536). For the polarized tests, the X shows a 5.1- and 2.2-fold excess of positively selected genes on the X chromosome at the *P* < 0.01 and 0.05 levels (*P*_FET_ ≤ 0.001; supplementary table S5, Supplementary Material online). The X and autosomes do not differ in the frequency of genes with excess nonsynonymous polymorphism (*P*_FET_ ≥ 0.550). For the unpolarized MK data, the estimate of the proportion of amino acid substitutions fixed by positive selection (α) is calculated using synonymous mutations as a neutral reference class, following Fay et al. (2002) and excluding singletons. The mean value of α for X-linked genes (0.240, CI_95_ = 0.199–0.282) is less than that for autosomal genes (0.359, CI_95_ = 0.344–0.373; table 2; but see below). For the polarized MK data, the opposite appears true: α for X-linked genes (0.362, CI_95_ = 0.305–0.418) is significantly greater than that for autosomal genes (0.211, CI_95_ = 0.181–0.240; table 3). These analyses show that X-linked genes are more likely to have individually significant histories of recurrent positive selection relative to autosomal genes and, at least for the polarized data, a greater fraction of nonsynonymous substitutions is beneficial on the X.

**Table 2:** Mean and 95% Confidence Intervals for the Estimated Proportion of Nonsynonymous Fixations on the X Chromosome (α_X_) and Autosomes (α_A_) That Are Driven by Positive Selection, Assuming Synonymous Sites Are Selectively Neutral

	*α*_X_		*α*_A_	
	Mean	CI_95_	Mean	CI_95_
Polarized	0.362	0.305–0.418	0.211	0.181–0.240
Unpolarized	0.240	0.199–0.282	0.359	0.344–0.373

**Table 3:** Mean and 95% Confidence Intervals for the Estimated Proportion of Fixations on the X Chromosome (α*_X_) and Autosomes (α*_A_) Driven by Positive Selection for a Variety of Sequence Classes, Assuming Short Introns Are Selectively Neutral

Sequence	α*_X_		α*_A_	
Class	Mean	CI_95_	Mean	CI_95_
Synonymous	0.372	0.319 to 0.418	0.277	0.258 to 0.295
Nonsynonymous	0.583	0.541 to 0.572	0.559	0.546 to 0.370
Intron	−0.132	−0.203 to −0.063	−0.055	−0.080 to −0.030
UTR	0.236	0.187 to 0.282	0.241	0.221 to 0.259

We tested for Gene Ontology (GO) category enrichment among genes having histories of recurrent adaptive protein evolution (Eden et al. 2009). Over the long *D. mauritiana–D. melanogaster* history of divergence (unpolarized MK data), the top genes function in reproduction, the nuclear pore complex (including the hybrid incompatibility gene, *Nup160*; Tang and Presgraves 2009), and the regulation of satellite DNA (supplementary table S6, Supplementary Material online). While not detected by formal GO analyses, we noted that 6/14 genes with roles in kinetochore function show evidence of recurrent positive selection, consistent with models of genetic conflict over centromeric drive (Henikoff et al. 2001; Malik and Henikoff 2001; Przewloka et al. 2007). During the recent history of the *D. mauritiana* lineage (polarized MK data), the top genes function in the female germline (e.g., female germ-line cyst formation), mRNA catabolism (e.g., nuclear-transcribed mRNA catabolic process, nonsense-mediated decay), chromosome biology, and the regulation of satellite DNAs (e.g., *dodeca-satellite-binding protein* and *topoisomerase* 2; supplementary table S7, Supplementary Material online).

We also estimated the proportion of substitutions attributable to positive selection using SI8–30 sites as a putatively neutral reference class (hereafter, α*), instead of synonymous sites (α), for the unpolarized data (table 3). On the X chromosome, nonsynonymous (0.583, CI_95_ = 0.541–0.619), synonymous (0.372, CI_95_ = 0.319–0.418), and UTR substitutions (0.236, CI_95_ = 0.187–0.282) all show evidence for substantial adaptive evolution, whereas intronic sequences do not (−0.132, CI_95_ = −0.203 to −0.063). Similarly, on the autosomes, nonsynonymous substitutions have the highest α* (0.559, CI_95_ = 0.546–0.572), followed by synonymous (0.277, CI_95_ = 0.258–0.295) and UTR (0.241, CI_95_ = 0.221–0.259) substitutions, whereas intronic substitutions show no evidence for adaptive evolution ( − 0.055, CI_95_ = −0.080 to −0.030). These analyses provide evidence for adaptive evolution at UTR sequences (Andolfatto 2005) and, surprisingly, at synonymous sites. The latter finding has three implications. First, if true, then α for unpolarized nonsynonymous substitutions (α = 0.240 and 0.359 for X and autosomes, respectively; see above) are likely to be underestimates (α* = 0.583 and 0.559 for X and autosomes, respectively). Second, the X and the autosomes show comparable α* estimates among all sequence classes except synonymous substitutions, for which α* is significantly higher on the X (0.372 vs. 0.277). This finding helps to explain why α for nonsynonymous substitutions on the X appeared lower than on the autosomes in the unpolarized MK data (see above). Third, the higher α* for synonymous sites on the X chromosome implies a greater efficacy of weak positive selection than on the autosomes. However, as X-linked UTRs do not show an elevated α* relative to autosomes, the elevated α* at X-linked synonymous sites would seem to be attributable to the biology of biased codon usage rather than a generally greater efficacy of selection on the X. Interestingly, X-linked genes maintain higher codon usage bias for preferred synonymous codons in *Drosophila* (Singh et al. 2005, 2008). The present results suggest that synonymous sites genome-wide may be adapting to new or shifting optimal codon preferences (Comeron and Kreitman 2002; DuMont et al. 2004). We cannot, however, exclude the possibility that the signal of adaptive evolution (or, part of it) results from a historical change in effective population size or functional constraints (Eyre-Walker 2002).

### Recent Selective Sweeps

The SweepFinder analyses of 10,576 ten-kilobase windows from ten *D. mauritiana* strains identify 152 windows (1.4%) with significantly aberrant SFS relative to their chromosomal background at the *P* < 0.05 level (fig. 1). Significant windows are overrepresented on the X chromosome (61) relative to the autosomes (91; χ^2 ^= 54.68, df = 1, *P* = 1.4 × 10^−13^). To characterize the distribution of polymorphism in these anomalous regions, we compared summaries of polymorphism (*π*), linkage disequilibrium (*Z_nS_*), and the SFS (using Tajima’s *D*) in the 152 aberrant windows with randomly selected windows using a simple randomization test. We performed 1,000 replicates randomly selecting 152 windows without replacement, divided among chromosome arms to match the distribution of aberrant windows. Anomalous windows have significantly lower *π* and significantly higher *Z_nS_* (*P* < 0.05). Although Tajima’s *D* is not different, the variance in Tajima’s *D* is significantly larger among anomalous windows (supplementary tables S8–S11, Supplementary Material online). For inferred sweep regions that are ≥20 kb, eight large sweeps have affected approximately 4.1% of the total map length of chromosome *X*; four large sweeps affect approximately 1.7% of the length of chromosome *2L*, four affect 0.8% of *2R*, five affect 0.4% of *3L*, and no sweeps ≥20 kb are detected on chromosome *3R* (table 4). Finally, the presence of these putative sweeps alone cannot account for the reduced *π*_X_/*π*_A_ ratio (table 1); when all putative sweep regions on the X are excluded, *π*_X_/*π*_A_ = 0.655 for the remaining X-linked sites.

**Table 4:** Recently Swept Regions of the *Drosophila mauritiana* Genome That Are Larger Than 20 kb

Chromosome	Coordinates	Length (kb)	cM
*2L*	1,445,000–1,468,000	23	0.095
*2L*	20,687,000–20,943,000	256	0.405
*2L*	22,406,000–22,635,000	229	0.362
*2L*	22,837,000–23,030,000	193	0.305
*2R*	1,390,000–1,576,000	186	0.294
*2R*	1,704,000–1,760,000	56	0.088
*2R*	3,064,000–3,087,000	23	0.143
*2R*	8,774,000–8,798,000	24	0.119
*3L*	7,000–128,000	121	0.399
*3L*	22,379,000–22,493,000	114	0.007
*3L*	22,687,000–22,879,000	192	0.012
*3L*	23,184,000–23,313,000	129	0.007
*3L*	23,562,000–23,610,000	48	0.003
*3L*	23,712,000–23,751,000	39	0.002
*X*	41,000–120,000	79	0.072
*X*	436,000–457,000	21	0.019
*X*	7,127,000–7,160,000	33	0.220
*X*	8,500,000–9,050,000	550	3.008
*X*	9,351,000–9,375,000	24	0.130
*X*	14,629,000–14,661,000	32	0.107
*X*	16,134,000–16,274,000	140	1.108
*X*	19,996,000–20,227,000	231	0.679

Note.—The sweep coordinates given are from the *mau12* genome assembly. The estimated size of the putative sweep region is provided in physical distance (kb) and centimorgans (True, Mercer, et al. 1996).

**Fig. 2.—:**
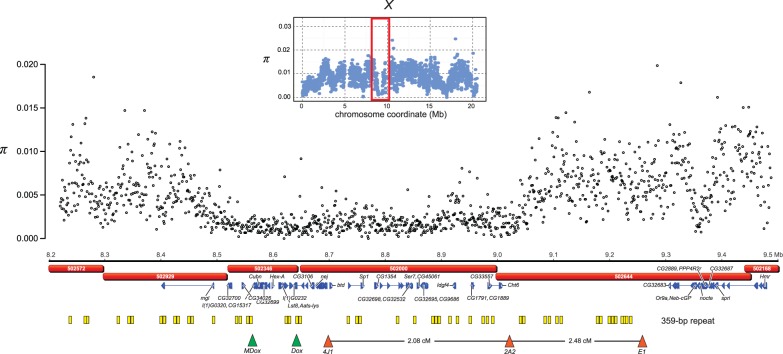
Nucleotide diversity (*π*) across a large selective sweep on the X chromosome of *D. mauritiana*. The red rectangle in the inset above the graph delimits the region of reduced polymorphism on the X chromosome. Unfilled circles plot nucleotide diversity in 1-kb windows between positions X: 8,500,000–9,050,000. The red bars below the physical position of 1-kb windows represent the scaffolds constituting this region in the de novo assembly. Below the scaffolds are blue triangles that depict the gene models for a representative group of genes in the putative sweep regions (the names are from the homologous annotations in the *D. melanogaster* genome). The yellow squares mark the positions of 359-like satellite DNAs. Below the gene models are orange-filled triangles that show the positions of the markers used by True, Mercer, et al. (1996) and the observed intervening recombination distances. Finally, the green triangles give the locations of the two Winters *sex-ratio* meiotic drive genes in the *D. simulans* genome.

The majority of putative selective sweeps on the autosomes occur in low-recombination centromere-proximal regions (fig. 1). However, several of the putative selective sweeps on the X chromosome affect unusually large physical and recombination distances in the middle of the chromosome (table 4). The largest putative selective sweep occurs between positions X:8,500,000–9,050,000 (fig. 2). Interestingly, this 3-cM sweep region is enriched for satellite DNAs (figs. 2 and 4 and see the following subsection) and spans the locations of the Winters *sex-ratio* genes, *MDox* and *Dox*, which have been shown to be part of a cryptic male meiotic drive system in *D. simulans* (Tao et al. 2007; Kingan et al. 2010). To assess the potential contribution of the Winters *sex-ratio* genes to this selective sweep, we assayed the presence of both *Dox* and *MDox* in 26 *D. mauritiana* strains, including six strains used for whole-genome sequencing (supplementary methods, Supplementary Material online). We find that *Dox* is present in only 23% (6/26) of these strains (supplementary fig. S2, Supplementary Material online) and therefore could not have caused the large selective sweep. (The reference strain, *mau12*, lacks the *Dox* gene.) *MDox*, however, appears fixed in *D. mauritiana* (26/26; supplementary fig. S3, Supplementary Material online). Thus, *MDox* remains a potential cause of the selective sweep.

**Fig. 3.—:**
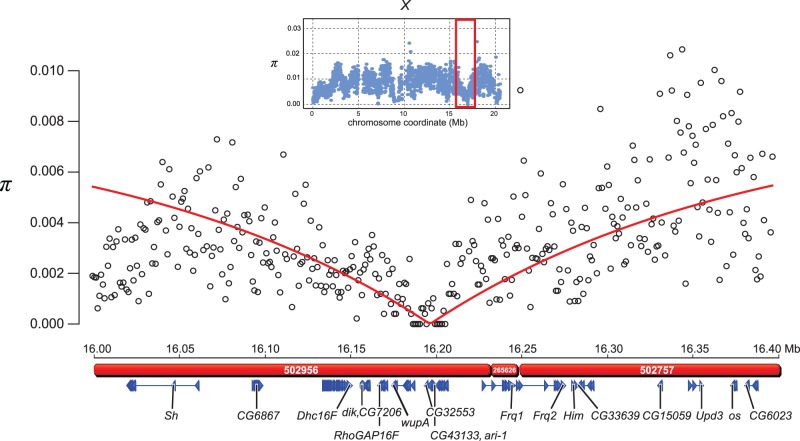
A canonical selective sweep on the *D. mauritiana* X chromosome spans more than 140 kb. The red rectangle in the inset above the graph delimits the region of reduced polymorphism on the X chromosome. Unfilled circles plot nucleotide diversity (*π*) in 1-kb windows and the red line plots the expected *π* under a simple hard sweep model (see text). The red bars below the physical position of the 1-kb windows represent the scaffolds in the de novo assembly. Below the scaffolds are blue triangles showing the gene models. A 20-kb region of near-complete homozygosity contains four genes: *wupA*, *CG32553*, *CG43133*, and *ari-1*.

A second large sweep in a high-recombination region occurs sweep between positions 16,134,000 and 16,274,000 on the X chromosome (fig. 1). For this putative sweep, we estimate the population-scaled selection intensity (*γ* = 2*N*_e_*s*). We assume that nucleotide diversity before the sweep was *π*_0_ = 0.0082 (the average for the X chromosome) and that the local per-base crossing over rate is 7.91 × 10^−6^ (True, Mercer, et al. 1996). We then predict nucleotide diversity at recombination distance *c*, using the equation

(1) $$\pi_{c}=\pi_{0}(1-\gamma^{-4c/s})$$

(Barton 2000). We fit the model to the data using a simple least-squares approach. The best-fitting model occurs when *γ* = 7.05 × 10^5^ (fig. 3). In this candidate sweep region, there is a 20-kb stretch of near-complete homozygosity in *D. mauritiana*. From our RNAseq data, we have annotated four genes in this 20-kb region and identified their homologs in *D. melanogaster*: *wupA*, *CG32553*, *CG43133*, and *ari-1*. Of these four genes, only *CG32553* and *CG43133* have nonsynonymous substitutions exclusively in the *D. mauritiana* sample (supplementary table S12, Supplementary Material online). Neither *CG32553* nor *CG43133* has any known function or phenotype in *D. melanogaster* and their products do not possess homology with any known proteins or conserved domains, although, in *D. melanogaster*, *CG32533* is expressed exclusively in the eye and *CG43133* in the eye-antennal disc. Nolte et al. (2013) raised the possibility that the HMS locus *Odysseus* (*OdsH*) is located within this sweep region; however, in our assembly, *OdsH* maps to a distal region 200 kb away.

Finally, there is another large putative selective sweep on chromosome X:7,127,000–7,160,000 (table 4 and fig. 1). In this 33-kb region, there is again a 20-kb interval of nearly complete homozygosity among the ten *D. mauritiana* strains. The RNAseq data indicate that six transcripts map to this 20-kb interval (data not shown), each with a homolog in *D. melanogaster*: *Hira*, *NELF-B*, *CG12155*, *Pdp*, *Rab39*, and *Tom40*. Interestingly, expression of the *Hira* gene is known to be associated with *Wolbachia*-induced cytoplasmic incompatibility in male *D. melanogaster* (Zheng et al. 2011).

### Genomic Distribution of Repetitive DNAs

We characterized the genomic distribution of five classes of repetitive DNA in the de novo assembly of the reference strain *mau12*: long terminal repeat (LTR) and non-LTR retrotransposons, DNA transposons, satellite DNAs, and microsatellites. These analyses disproportionately survey the euchromatic portions of the genome, because heterochromatic regions can be underrepresented in the assembly. We find that the densities of all classes of repetitive DNA are significantly heterogeneous among chromosome arms (χ^2 ^= 72.30, df = 3, *P* < 10^−5^; supplementary table S13, Supplementary Material online). Curiously, DNA transposons and both classes of retroelements are underrepresented on chromosome arm *3R* and overrepresented on *2R*. The other classes of repetitive DNAs are all overrepresented exclusively on the X chromosome; Although significant heterogeneity does exist among autosomal arms, all four arms show a dearth of repetitive DNAs relative to the X. The X chromosome has an approximately 1.4-fold excess of microsatellites (0.035% of the sequence) and low complexity (0.005%) repeats relative to the autosomes (0.02% and 0.003%, respectively; see also Bachtrog et al. 1999). And, strikingly, the X chromosome has a 7.5-fold excess of satellite DNA (*P* < 10^−5^; 0.5%) relative to the autosomes (0.04%; see also Gallach 2014). Although satellite DNAs are typically organized as large blocks in pericentric heterochromatin, those detected here correspond to satellite DNA islands in the euchromatin (Kuhn et al. 2012). As satellite DNAs are repetitive and organized into arrays longer than can be spanned by short-read sequences, our analyses probably detect only the edges of satellite DNA islands and thus underestimate the total amount of satellite DNA.

**Fig. 4.—:**
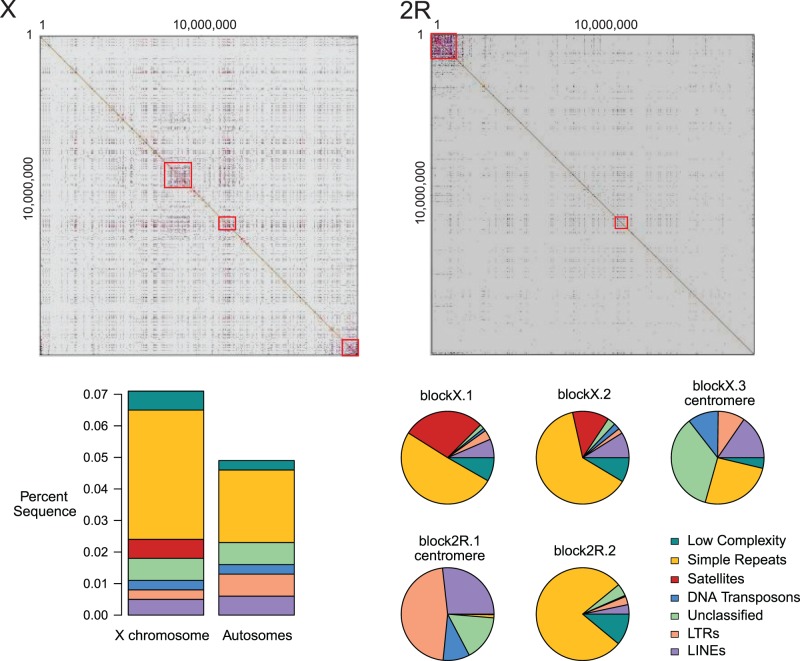
Repeat content differs on the X and autosomes. In the top row, dot plots of the X chromosome and a representative autosomal arm (*2R*) show an enrichment of repetitive sequence on the X, which is organized into blocks of high repeat density. The bottom left panel shows the Percent repetitive sequence for the X chromosome compared with the average across the major autosomal arms. Finally, the bottom right panel shows the proportion of the different repeat classes in the highlighted repeat blocks on chromosomes *X* and *2R*.

Dot plots highlight the heterogeneity in repetitive DNA densities along chromosome arms, as well as the qualitative difference in repetitive DNA densities between the X and autosomes (fig. 4). On chromosome arm *2R*, the pericentromeric proximal region is enriched for LTR retroelements, non-LTR retroelements, and DNA transposons. On the X, there are large megabase-scale regions in the middle of the chromosome arm with high densities of satellite DNAs, microsatellites, and low complexity sequences. The two most conspicuous concentrations of repetitive DNAs occur between coordinates X:8,235,022–9,591,378 (block X.1) and X:11625913–12594739 (block X.2). The coordinates of the first region coincide with those of the distal large 550-kb sweep region on the X (fig. 2 and table 4). Slightly more than half of the satellite DNA occurring in this region have homology with the 359-bp satellite (supplementary table S14, Supplementary Material online). Although there is evidence that the *sex-ratio* meiotic drive genes are not involved in this large selective sweep, the possibility remains that selfish satellite DNAs may play a role in eliminating variation over this large genomic region.

## Discussion

Our survey of diversity and divergence from ten high-quality *D. mauritiana* genomes shows that the X chromosome bears more signatures of recent and recurrent positive natural selection than do the autosomes. The observed low X/A ratio of diversity cannot be easily explained by a recent population bottleneck (cf. Pool and Nielsen 2008). Our genome-wide analyses confirm that *D. mauritiana* harbors a surprisingly high level of nucleotide diversity for an island endemic species. Furthermore, the genome-wide SFS shows a mild excess of low frequency polymorphisms, a pattern that is more consistent with weak population expansion and/or functional constraints. In our data, there is no clear evidence for a recent recovery from a severe population bottleneck (see also Kliman et al. 2000). Instead, despite having what must be a smaller census population size than, say, the cosmopolitan species *D. melanogaster*, *D. mauritiana* maintains more diversity overall and, hence, a relatively large effective population size. Finally, the low X/A ratio of diversity cannot be attributed to greater functional constraints on the X, because the SFS is comparable between the X and the autosomes for all sequence classes (table 1), and the X shows higher divergence from *D. melanogaster* than do the autosomes. The disparity between X/A diversity versus divergence thus implies that positive natural selection acts more frequently on the X chromosome than the autosomes.

Our genome-wide scan for anomalous SFS confirms that the X chromosome of *D. mauritiana* has experienced more recent selective sweeps than have the autosomes. In general, these X chromosome sweeps are larger and produce more aberrant SFS than those on the autosomes (fig. 1 and table 4). The excess of recent sweeps on the X cannot be attributed to adaptation from standing genetic variation, which causes difficult-to-detect “soft” sweeps (Hermisson and Pennings 2005; Teshima et al. 2006), and is expected to progress more slowly on the X chromosome than the autosomes (Orr and Betancourt 2001). Instead, these findings suggest that the X has experienced more classic “hard” selective sweeps resulting from new beneficial mutations. A higher rate of adaptive evolution on the X is expected when new beneficial mutations are, on average, at least partially recessive (Charlesworth et al. 1987; Vicoso and Charlesworth 2009). This faster X model of adaptive molecular evolution may explain some of the excess divergence (Charlesworth et al. 1987) and reduced diversity on the X chromosome (Betancourt et al. 2004).

The relative abundance of hitchhiking effects on the X chromosome may be attributable to selfish genetic elements. Sex chromosomes are susceptible to the invasion and spread of multilocus meiotic drive elements (Hurst and Pomiankowski 1991), and these can cause patterns indistinguishable from classic selective sweeps (e.g., Derome et al. 2008). The distal 3-cM sweep valley on the *D. mauritiana* chromosome *X* spans the *MDox* locus, which may be the original distorter element of the Winters *sex-ratio* system (Tao et al. 2007; Kingan et al. 2010). Although additional data clearly show that the *Dox* locus cannot be the target of selection in this case, the progenitor *MDox* locus remains a viable explanation for this sweep. However, this large distal sweep also spans a particularly high density of 359-like satellite DNAs, which may also spread by manipulating transmission (Ferree and Prasad 2012; Gallach 2014). In primates, selective sweep regions on the X chromosome are also enriched for ampliconic genes (Nam et al. 2014), which may similarly be involved in conflict over transmission. Finally, our genome-wide MK analyses show that genes with individually significant evidence for recurrent adaptive protein evolution are overrepresented on the X chromosome and, separately, tend to be enriched for functions in reproductive biology and/or chromosome biology, suggestive of frequent genetic conflict (e.g., satellite DNA-binding proteins, nuclear pore proteins, kinetochore proteins).

Ours is the second population genomics analysis of *D. mauritiana*. The first used a “Pool-seq” strategy, sequencing 107 isofemale lines pooled to approximately 110-fold depth overall (Nolte et al. 2013). This shallow coverage of a deep sample is complementary to our deep coverage of a phased, shallow sample. Indeed, we detect the same faster X divergence, the same prominent selective sweeps on the X chromosome, and comparable sets of genes as targets of past recurrent adaptation. There are however large differences in our estimates of basic summary statistics. Despite a smaller sample, our estimates of *π* are 36% and 44% higher for the X and the autosomes, respectively. Conversely, our estimates of Tajima’s *D* are 3.7- and 5.2-fold smaller than those of Nolte et al. (2013) for the X and the autosomes, respectively (e.g., Tajima’s *D* = −1.79 vs. −0.33 for the autosomes; table 1). As noted by Nolte et al., the Pool-seq approach is biased toward an excess of rare variants, as sequencing errors are not readily distinguishable. Similarly, Lynch et al. (2014) estimated that the sequencing depth for Pool-seq must be 5-10× the sample size to infer low frequency alleles accurately. These comparisons suggest that Pool-seq can be useful for estimating net divergence and characterizing gross patterns of polymorphism, but not for the high-quality single nucleotide polymorphism calling necessary for accurate parameter estimation (Cutler and Jensen 2010).

The availability of population genomic data in *D. mauritiana* has two impacts on speciation genetics. First, for the past decade, evidence for faster X evolution in *Drosophila* has been equivocal, making it a doubtful contributor to the large X-effect for HMS (Presgraves 2008b). However, the present analyses, and others (Begun et al. 2007; Langley et al. 2012; Llopart 2012; Mackay et al. 2012; Meisel et al. 2012), firmly establish the fact of faster X evolution, although its biological basis remains to be determined (Meisel and Connallon 2013). Our analyses, and others’ (Begun et al. 2007; Nolte et al. 2013), are also consistent with the notion that genetic conflict is a major driver of molecular evolution, especially on the X chromosome (Frank 1991; Hurst and Pomiankowski 1991; Meiklejohn and Tao 2010). Second, the discovery that the *D. mauritiana* allele of *Odysseus* causes male sterility in a *D. simulans* genetic background (Ting et al. 1998) was a major step toward understanding the molecular biology of HMS. But identifying a panel of X-linked HMS factors is necessary to draw broad conclusions about the molecular basis of the large X-effect. Population genomics resources in *D. mauritiana* and *D. simulans* (Begun et al. 2007; Rogers et al. 2014) will empower genetic mapping efforts and accelerate identification of more genes involved in HMS and other species differences.

## Supplementary Material

Supplementary methods, figures S1–S3, and tables S1–S16 are available at *Genome Biology and Evolution* online (http://www.gbe.oxfordjournals.org/).

Supplementary Data
