# Supplementary material for: Genome Diversity and Divergence in Drosophila mauritiana: Multiple Signatures of Faster X Evolution
Source: Genome Biol Evol. 2014 Sep 9;6(9):2444–58. doi: 10.1093/gbe/evu198 (PMC4202334; doi:10.1093/gbe/evu198)
Supplement: Supplementary Data [file supp_6_9_2444__index.html]

Genome Diversity and Divergence in Drosophila mauritiana: Multiple Signatures of Faster X Evolution — Supplementary Data 

# Genome Diversity and Divergence in *Drosophila mauritiana*: Multiple Signatures of Faster X Evolution

## Supplementary Data

files

**Files in this Data Supplement:**

- Supplementary Data - pdf file
