# Supplementary material for: Genome Diversity and Divergence in Drosophila mauritiana: Multiple Signatures of Faster X Evolution
Source: Genome Biol Evol. 2014 Sep 9;6(9):2444–58. doi: 10.1093/gbe/evu198 (PMC4202334; doi:10.1093/gbe/evu198)

Supplementary materials for

Genomic diversity and divergence in *Drosophila mauritiana*: multiple signatures of faster X evolution

Daniel Garrigan[1,*], Sarah B. Kingan[1], Anthony J. Geneva[1], Jeffrey P. Vedanayagam[1], and Daven C. Presgraves[1]

[1]Department of Biology, University of Rochester, Rochester, New York, USA

[*]Corresponding author: dgarriga@ur.rochester.edu

Methods

The Winters *sex-ratio* (SR) is one of three X-linked meiotic drive system in *Drosophila simulans* (Tao Araripe et al. 2007). This system is comprised of a compound drive locus: *Distorter on the X* (*Dox*) and *Mother of Dox* (*MDox*) and an autosomal suppressor, *Not much yang* (*Nmy*). *Dox* originated as a duplicate copy of *MDox* and *Nmy* is a retrotransposed copy of *Dox* (Tao, Araripe et al. 2007). A functional copy of *Nmy* exists in both *D. simulans* and *D. mauritiana*, and is capable of suppressing drive, indicating that the Winters SR evolved in the *D. simulans* clade ancestor. These genes were previously shown to bear a signature of a selective sweep in *D. simulans* (Kingan et al. 2010). Here we characterize polymorphism at *Dox* and *MDox* in a larger sample of isofemale *D. mauritiana* lines (see main paper methods for collection details) to ask whether the Winters driver may be the target of selection.

Genomic DNA was extracted from single males using a modified protocol of the Wizard Genomic DNA Purification Kit from Promega. Polymerase chain reaction was performed using

Takara LA Taq polymerase following the manufacturer's instructions with a 5 minute extension time. PCR products were cleaned prior to sequencing using exoSAPit (USB). Previously published PCR primers for the *Dox* and *MDox* regions were used, which amplified complete genes as well as flanking sequence (Tao, Araripe, et al. 2007; Tao, Masly, et al. 2007; Kingan, et al. 2010; see supplementary table S14). Internal sequencing primers were designed using Primer3Plus (Untergasser, et al. 2007) and Amplify (Engels 2005); sequences are available upon request. Sequencing was performed on an ABI3730 capillary sequencer according to manufacturer's protocols; sequences were edited using Sequencher v.4.8 (Gene Codes Corp.). Many of the haplotypes from the *Dox* region have a series of identical or nearly identical copies of a 359-bp satellite DNA repeat element (Tao, Araripe, et al. 2007), which is related to the satellite block, *Zhr* (Ferree and Barbash 2009), which complicated the assembly of contigs. We digested the amplified PCR fragments with BslI, and compared the restriction fragment profile with that predicted from our contig to ensure the correct number of repeat elements were included in our alignments. 2× sequencing coverage was obtained for all samples, forward and reverse reads when possible, but in many cases, the lack of unique priming sites due to the tandemly arrayed repeat elements only allowed for 2× coverage in the same direction.

Alignments of sequences for each gene region, as well as between homologous regions of *Dox* and *MDox* were executed by eye using annotated sequence elements as "anchors" for the alignment. In some cases, the bl2seq program of BLAST was used for pair wise alignments (Tatusova and Madden 1999). To insure a proper alignment of each region, we performed a phylogenetic analysis of the 359-bp satellite elements for each gene to assign homology among repeats. For each locus, we extracted the repeat sequences from each sampled isofemale line and aligned the repeats by eye.

Results

At the *MDox* region, we sample 26 chromosomes and compile a 5,526-bp alignment of the sequences (**supplementary table S16**). We infer that *MDox* is fixed in *D. mauritiana* because all 26 samples contain the gene insertion. We observe copy number variation in the 359-bp satellite elements that flank the *MDox* gene: copy number ranges from one to four tandemly arrayed repeats with most samples having two elements (**supplementary fig. S3**). **Fig. S3b** shows the neighbor-joining tree that was used to create an accurate alignment of the sequenced region (we obtained a similar trees using parsimony, not shown). In addition to the *D. mauritiana* lines sampled in this study, we include 69 previously sequenced *D. simulans* samples (Kingan, et al. 2010). We observe four clusters of repeat sequences with more than 75% bootstrap support and define these as *MDox* repeat types one through four.

At the *Dox* region, we sample the same 26 *D. mauritiana* chromosomes and compile an alignment of 8,503-bp. The ancestral *Dox[null]* allele, which lacks the *Dox* gene insertion, is more common than the derived allele, and is found in 20 of the sampled chromosomes (~77%) (Kingan, et al. 2010). One of the sampled strains (*R10*) has a ~3.2-kb deletion that is part of the *Dox* gene. Furthermore, we observe extensive copy number variation in the 359-bp satellite in *D. mauritiana*, with the number of elements ranging from four to seven (**supplementary fig. S2**). **Supplementary fig. S2b** also shows the neighbor-joining tree that was used to assemble an accurate alignment of the *Dox* region for our 26 *D. mauritiana* samples, as well as our previously collected *D. simulans* samples (Kingan, et al. 2010). We observe five clusters of elements with more than 56% bootstrap support and define these as *Dox* repeat types one through five. (No quantitative inferences are made about the evolution of repeat types from these

trees— they are simply used to compile the best alignment of the sequence data by clustering the most closely related repeat elements.)

Literature Cited

Engels WR. 2005. Amplify. Version 3.14: University of Wisconsin. http://engels.genetics.wisc.edu/amplify/.

Ferree PM, Barbash DA. 2009. Species-specific heterochromatin prevents mitotic chromosome segregation to cause hybrid lethality in *Drosophila*. PLoS Biol 7:e1000234.

Kingan S, Garrigan D, Hartl D. 2010. Recurrent selection on the Winters *sex-ratio* genes in *Drosophila simulans*. Genetics 184:253-265.

Tao Y, Araripe L, Kingan SB, Ke Y, Xiao H, Hartl DL. 2007. A *sex-ratio* meiotic drive system in *Drosophila simulans*. II: an X-linked distorter. PLoS Biol 5:e293.

Tao Y, Masly JP, Araripe L, Ke Y, Hartl DL. 2007. A *sex-ratio* meiotic drive system in *Drosophila simulans*. I: an autosomal suppressor. PLoS Biol 5:e292.

Tatusova TA, Madden TL. 1999. BLAST 2 Sequences, a new tool for comparing protein and nucleotide sequences. FEMS Microbiol Lett 174:247-250.

Untergasser A, Nijveen H, Rao X, Bisseling T, Geurts R, Leunissen JA. 2007. Primer3Plus, an enhanced web interface to Primer3. Nucleic Acids Res 35:W71-74.

Table S1

Statistics describing the *de novo* assembly of the *mau12* genome.

measure	value
assembly size	124,206,178
number of contigs (> 1000-bp)	2,759
largest contig	2,989,439
N50	542,067
N75	235,193

Table S2

The number of contigs used to construct the syntenic pseudo-chromosomes. The length of the pseudo-chromosomes does not include any ambiguous bases that were used to fill gaps.

chromosome	contigs	length (bp)
2L	123	21,521,355
2R	151	18,987,217
3L	120	21,693,048
3R	59	25,652,786
X	150	17,830,043
total	603	105,684,449

Table S3

Details of the libraries sequenced for each strain of *Drosophila*. The penultimate column lists the number of reads mapping to the *mau12* reference genome after quality filtering.

strain	species	length (bp)	insert (bp)	mapped reads ($x10^6$)	accession
mau12	*mauritiana*	86	200	37.311	SRR483621
	mauritiana	100	3000	122.031	n/a
R23	*mauritiana*	76	350	57.315	n/a
	mauritiana	76	350	63.366	n/a
R31	*mauritiana*	76	350	47.173	n/a
	mauritiana	76	350	55.987	n/a
R32	*mauritiana*	76	350	58.338	n/a
	mauritiana	76	350	66.923	n/a
R39	*mauritiana*	76	350	55.664	n/a
	mauritiana	76	350	67.235	n/a
R41	*mauritiana*	76	350	60.61	n/a
	mauritiana	76	350	64.477	n/a
	mauritiana	76	350	61.971	n/a
R44	*mauritiana*	76	350	61.604	n/a
	mauritiana	76	350	67.161	n/a
R56	*mauritiana*	76	350	58.934	n/a
	mauritiana	76	350	68.625	n/a
R61	*mauritiana*	76	350	60.55	n/a
	mauritiana	76	350	62.871	n/a
	mauritiana	76	350	59.72	n/a
R8	*mauritiana*	76	350	61.991	n/a
	mauritiana	76	350	54.09	n/a
MD063	*simulans*	76	350	51.55	SRR1200800
	simulans	76	350	48.003	n/a
w^{501}	*simulans*	100	500	37.974	SRR520334
y; cn bw; sp	*melanogaster*	146	300	46.061	SRR306626
	melanogaster	146	300	57.431	SRR306627
	melanogaster	146	300	60.617	SRR306628

Table S4

The 13 strains of *Drosophila* used in this study. Also included are the median read depth for each aligned position and the percent of *mau12* reference genome covered in each of the ten lines of *D. mauritiana*, two lines of *D. simulans*, and the reference strain of *D. melanogaster*, after quality filtering.

strain	species	depth	% covered
mau12	*mauritiana*	125	99.48
R23	*mauritiana*	84	98.83
R31	*mauritiana*	72	98.86
R32	*mauritiana*	87	98.84
R39	*mauritiana*	86	98.84
R41	*mauritiana*	130	98.86
R44	*mauritiana*	89	98.80
R56	*mauritiana*	90	98.86
R61	*mauritiana*	130	98.78
R8	*mauritiana*	81	98.86
MD063	*simulans*	70	98.18
w^{501}	*simulans*	34	97.91
y; cn bw; sp	*melanogaster*	110	94.13

Table S5

Comparison of counts of genes on the X chromosome (X) and autosomes (A) with significant McDonald-Kreitman tests. Positive (pos.) and negative (neg.) gene categories are genes that show significant departure from neutral expectations in the direction of positive selection (excess nonsynonymous divergence) or negative selection (reduced nonsynonymous divergence), respectively, at the indicated significance cut-off ($P < 0.05$ or $P < 0.01$). Fisher's Exact Test (FET) *P*-value compares X-linked and autosomal counts for significant genes versus all genes tested.

test	gene category	X	A	FET *P*
polarized	pos ($P < 0.05$)	26	65	0.0011
	neg ($P < 0.05$)	4	22	1.0000
polarized	pos ($P < 0.01$)	11	12	0.0002
	neg ($P < 0.01$)	0	5	1.0000
	All tested	1,690	9,349	
unpolarized	pos ($P < 0.05$)	60	357	0.5359
	neg ($P < 0.05$)	45	222	0.5501
unpolarized	pos ($P < 0.01$)	33	111	0.0200
	neg ($P < 0.01$)	14	75	1.0000
	all tested	1,757	9,523	

Table S6

Top Gene Ontology (GO) process, function, and component terms enriched among genes with evidence of recurrent adaptive evolution in the history of the *D. mauritiana* and *D. melanogaster* lineages (unpolarized MK test).

GO category	description
Process	
GO:0044702	single organism reproductive process
GO:0022414	reproductive process
GO:0048610	cellular process involved in reproduction
GO:0044767	single-organism developmental process
GO:0032502	developmental process
GO:0007276	gamete generation
GO:0022412	cellular process involved in reproduction in multicellular organism
GO:0032862	activation of Rho GTPase activity
GO:0032863	activation of Rac GTPase activity
GO:0007568	aging
Function	
GO:0003676	nucleic acid binding
GO:0045502	dynein binding
GO:0004534	5'-3' exoribonuclease activity
GO:0008574	plus-end-directed microtubule motor activity
GO:0003723	RNA binding
GO:0008270	zinc ion binding
GO:0003696	satellite DNA binding
GO:0080023	3R-hydroxyacyl-CoA dehydratase activity
GO:0008409	5'-3' exonuclease activity
Component	
GO:0044611	nuclear pore inner ring
GO:0038201	TOR complex
GO:0032991	macromolecular complex
GO:0044427	chromosomal part
GO:0044428	nuclear part
GO:0005737	cytoplasm
GO:0043228	non-membrane-bounded organelle
GO:0043232	intracellular non-membrane-bounded organelle

Table S7

Top Gene Ontology (GO) process, function, and component terms enriched among genes with histories of recurrent adaptive evolution in the *D. mauritiana* lineage (polarized MK test).

GO category	description
Process	
GO:0007276	gamete generation
GO:0000184	nuclear-transcribed mRNA catabolic process, nonsense-mediated decay
GO:0016071	mRNA metabolic process
GO:0030727	germarium-derived female germ-line cyst formation
GO:0048135	female germ-line cyst formation
GO:0016070	RNA metabolic process
GO:0000956	nuclear-transcribed mRNA catabolic process
GO:0010629	negative regulation of gene expression
GO:0006402	mRNA catabolic process
GO:0000291	nuclear-transcribed mRNA catabolic process, exonucleolytic
Function	
GO:0009055	electron carrier activity
GO:0003696	satellite DNA binding
GO:0017056	structural constituent of nuclear pore
GO:0004532	exoribonuclease activity
GO:0016896	exoribonuclease activity, producing 5'-phosphomonoesters
Component	
GO:0005700	polytene chromosome
GO:0043228	non-membrane-bounded organelle
GO:0043232	intracellular non-membrane-bounded organelle
GO:0005604	basement membrane
GO:0000151	ubiquitin ligase complex
GO:0044420	extracellular matrix part
GO:0031461	cullin-RING ubiquitin ligase complex
GO:0005694	chromosome
GO:0044428	nuclear part
GO:0044427	chromosomal part
GO:0044451	nucleoplasm part
GO:0044611	nuclear pore inner ring

Table S8

Mean and median values of *D. mauritiana* nucleotide diversity (π) calculated for the 152 10-kb windows from SweepFinder with $P < 0.05$ and 152 randomly drawn windows sorted by chromosome. *P*-values are the probability that SweepFinder windows have lower π based on 1000 bootstrap resampling replicates.

		SweepFinder windows		random windows			
chrom	n windows	mean	median	mean	0.025 CI	0.975 CI	*P*-value
2L	56	0.0051	0.0028	0.0143	0.0123	0.0201	< 0.001
2R	19	0.0071	0.0054	0.0127	0.0112	0.0147	< 0.001
3L	10	0.0068	0.0062	0.0139	0.0115	0.0162	< 0.001
3R	6	0.0060	0.0059	0.0125	0.0101	0.0152	< 0.001
X	61	0.0023	0.0018	0.0085	0.0077	0.0093	< 0.001
aut.	91	0.0057	0.0041	0.0138	0.0124	0.0174	< 0.001

Table S9

Mean and median values of sequence divergence from *D. melanogaster* (average pairwise Jukes-Cantor divergence) calculated for the 152 10-kb windows from SweepFinder with $P < 0.05$ and 152 randomly drawn windows sorted by chromosome. *P*-values are the probability that SweepFinder windows have higher divergence based on 1000 bootstrap resampling replicates.

		SweepFinder windows		random windows			
chrom	n windows	mean	median	mean	0.025 CI	0.975 CI	*P*-value
2L	56	0.0623	0.0617	0.0425	0.0406	0.0445	< 0.001
2R	19	0.0563	0.0564	0.0410	0.0378	0.0444	< 0.001
3L	10	0.0407	0.0413	0.0417	0.0371	0.0468	0.592
3R	6	0.0493	0.0465	0.0410	0.0358	0.0470	0.013
X	61	0.0535	0.0547	0.0457	0.0438	0.0477	< 0.001
aut.	91	0.0577	0.0566	0.0420	0.0405	0.0435	< 0.001

Table S10

Mean and median values of Tajima's *D* (*TD*) for *D. mauritiana* calculated for the 152 10-kb windows from SweepFinder with $P < 0.05$ and 152 randomly drawn windows sorted by chromosome. *P*-values are the probability that SweepFinder windows have lower *TD* based on 1000 bootstrap resampling replicates.

		SweepFinder windows		random windows			
chrom	n windows	mean	median	mean	0.025 CI	0.975 CI	*P*-value
2L	56	−0.1811	−0.1079	−0.2887	−0.3500	−0.2298	0.005
2R	19	−0.2784	−0.3205	−0.4331	−0.5409	−0.3217	0.013
3L	10	−0.3823	−0.3602	−0.2211	−0.3803	−0.0460	0.952
3R	6	−0.6639	−0.6393	−0.3917	−0.5832	−0.1801	0.988
X	61	−0.4690	−0.5440	−0.5298	−0.5991	−0.4541	0.079
aut.	91	−0.2553	−0.2309	−0.3181	−0.3684	−0.2677	0.024

Table S11

Mean and median values of Z_{nS} (average pairwise r^2) for *D. mauritiana* calculated for the 152 10-kb windows from SweepFinder with $P < 0.05$ and 152 randomly drawn windows sorted by chromosome. *P*-values are the probability that SweepFinder windows have higher Z_{nS} based on 1000 bootstrap resampling replicates.

		SweepFinder windows		random windows			
chrom	n windows	mean	median	mean	0.025 CI	0.975 CI	*P*-value
2L	56	0.1647	0.1490	0.1364	0.1321	0.1417	< 0.001
2R	19	0.1333	0.1324	0.1245	0.1200	0.1310	0.024
3L	10	0.1391	0.1408	0.1371	0.1292	0.1484	0.270
3R	6	0.1730	0.1343	0.1310	0.1210	0.1437	0.006
X	61	0.2113	0.1457	0.1283	0.1223	0.1393	< 0.001
aut.	91	0.1559	0.1419	0.1336	0.1302	0.1374	< 0.001

Table S12

Polymorphism in the four genes in the putative sweep region X:16800000-17100000. Four nonsynonymous mutations are present and only three of those have arisen exclusively in the *D. mauritiana* lineage (one in the *CG32553* gene and two in the *CG43133* gene).

gene	*wup-A*	*CG32553*		*CG43133*					*ari-1*		
codon	133	27	35	26	31	47	60	95	102	127	380
mel	GAT	GCA	AAG	TTG	TAT	GCC	TTA	ATC	ACT	TCA	AAC
sim	...	...	CG.	...	...	...	...	...	...	...	...
R8	..C	.T.	C..	C..	..C	..A	A..	..G	..A	..G	..T
mau12	..C	.T.	C..	C..	..C	..A	A..	..G	..A	..G	..T
R23	..C	.T.	C..	C..	..C	..A	A..	..G	..A	..G	..T
R31	...	.T.	C..	C..	..C	..A	A..	..G	..A	..G	..T
R32	..C	.T.	C..	C..	..C	..A	A..	..G	..A	..G	..T
R39	...	.T.	C..	C..	..C	..A	A..	..G	..A	..G	..T
R41	..C	.T.	C..	C..	..C	..A	A..	..G	..A	..G	..T
R44	..C	.T.	C..	C..	..C	..A	A..	..G	..A	..G	..T
R56	..C	.T.	C..	C..	..C	..A	A..	..G	..A	..G	..T
R61	..C	.T.	C..	C..	..C	..A	A..	..G	..A	..G	..T
Change	syn	non	non	syn	syn	syn	non	non	syn	syn	syn
Amino Acid	D	A→V	K→Q	I	Y	A	L→I	I→M	T	S	N
			Q→R*								

*First nonsynonymous substitution occurred in simulans clade ancestor, second substitution in the *D. simulans* lineage.

Table S13

Percent masked sequence by chromosome arm for seven different repeat classes.

repeat class	*2L*	*2R*	*3L*	*3R*	*X*
LINES	0.0061	0.0087	0.0061	0.0029	0.0047
LTR elements	0.0097	0.0104	0.0064	0.0017	0.0023
DNA transposons	0.0043	0.0037	0.0028	0.0015	0.0026
unclassified	0.0076	0.0079	0.0061	0.0039	0.0065
satellites	0.0005	0.0004	0.0003	0.0005	0.0052
simple repeats	0.0174	0.0211	0.0215	0.0230	0.0354
low complexity	0.0027	0.0028	0.0032	0.0031	0.0051

Table S14

Identity of satellite DNA repeats present in block X.1 (see **fig. 3** from main text).

satellite	count	mean divergence
Sim359	45	15%
SAR_DM	26	20%
SAR2_DM	14	20%
SAT-1_Dsim	1	20%

Table S15

The sequences of PCR primers used to amplify the Winters *sex-ratio* genes in *D. mauritiana*.

name	sequence
CG32702_SR6_F1 (*MDox*)	GTAACGATGTGTACGCGCTTTGAGGTG
CG32702_SR6_R1 (*MDox*)	GGTCGCGGATTCACATTGCTCTATACC
DoxF4	AAGCAGTTCCCAAAGAAAGAGAGCAGAGCAACT
DoxR4	CCAGCTCAAAACCACAGGGAGACACTGTACATA

Table S16

Drosophila mauritiana strains sequences in this study.

strain	*Dox* genotype	*MDox* genotype
*mau12**	*Dox[null]*	*MDox*
R2	*Dox*	*MDox*
R3	*Dox[null]*	*MDox*
R4	*Dox[null]*	*MDox*
R6	*Dox[null]*	*MDox*
R7	*Dox*	*MDox*
*R8**	*Dox[null]*	*MDox*
R9	*Dox*	*MDox*
R10	*Dox[del]***	*MDox*
R11	*Dox[null]*	*MDox*
R12	*Dox[null]*	*MDox*
R13	*Dox[null]*	*MDox*
R16	*Dox*	*MDox*
R17	*Dox*	*MDox*
R18	*Dox[null]*	*MDox*
R19	*Dox[null]*	*MDox*
R20	*Dox[null]*	*MDox*
R22	*Dox[null]*	*MDox*
*R23**	*Dox[null]*	*MDox*
R26	*Dox[null]*	*MDox*
R27	*Dox[null]*	*MDox*
R30	*Dox[null]*	*MDox*
*R31**	*Dox[null]*	*MDox*
*R32**	*Dox[null]*	*MDox*
R35	*Dox[null]*	*MDox*
R38	*Dox[null]*	*MDox*
*R39**	*Dox[null]*	*MDox*

*Present in the population genomic dataset.

See **fig. S3 and supplementary text.

Supplementary Figure Legends

FIG. S1.— Distribution of the average pairwise nucleotide distance (π), the average Jukes-Cantor corrected distance from *D. melanogaster* (d_{mel}), and the summary of the site frequency spectrum, Tajima's *D* (*TD*), by chromosome for the whole genome (10-kb windows) and five different sequences classes. Box plots that are different colors have significantly different distributions (Mann-Whitney *U*, $P < 0.05$ after correcting for 10 pairwise comparisons).

FIG. S2.— Illustration of the alignment of *Dox* locus for *D. mauritiana* (above) and *D. simulans* (below). The 359-bp satellite DNA repeats are shown as rectangles. The colors correspond to repeat types based on the phylogenetic analysis; grey repeat types are present in only *Dox[null]* haplotypes of *D. simulans* and do not form a distinct clade (Panel B). "Type Sample" refers to the identity of one of the isofemale lines with the haplotype shown. Frequency is the number of times the haplotype appears in the sample. Length is the total length of the sequenced haplotype, which included repeats and flanking sequence. The *Dox* insertion is shown as a black triangle and is not drawn to scale. Gaps introduced into the alignment are shown as dashed lines. B) Bootstrap consensus neighbor-joining tree based on Jukes-Cantor genetics distance. The tree is rooted with *D. melanogaster* repeat elements from the *Dox* locus. Numbers at nodes are bootstrap values (values not shown are less than 40). Colors indicate repeat types as in Figure 3.4. "Tu" samples are *D. simulans*, "R" samples are *D. mauritiana*. Repeat elements for each sample are numbered in order (e.g., "R8 rep 7" and "R3 rep 5" are homologous and are both type 5 shown as purple). Repeat elements that contain the *Dox* insertion were concatenated and are indicated with an asterisk.

FIG. S3.— Illustrative alignment of the *MDox* locus for *D. mauritiana* (above) and *D. simulans* (below). The 359-bp satellite DNA repeats are shown as rectangles. Colors correspond to repeat

types based on the phylogenetic analysis; grey repeat types area present in only *MDox[null]* haplotypes of *D. simulans* and do not form a distinct clade (Panel B). “Type Sample” refers to the identity of one of the isofemale lines with the haplotype shown. Frequency is the number of times the haplotype appears in the sample. Length is the total length of the sequenced haplotype. The *MDox* insertion is shown as a black triangle and is not drawn to scale. Gaps introduced into the alignment are shown as dashed lines. B) Bootstrap consensus neighbor-joining tree based on Jukes-Cantor genetics distance. The tree is rooted with *D. melanogaster* repeat elements from the *Dox* locus. Numbers at nodes are bootstrap values (values at not shown are less than 40). Colors indicate repeat type as in Panel A. “Tu” samples are *D. simulans*, “R” samples are *D. mauritiana*. Repeats elements for each sample are numbered in order (e.g., “R18 rep 4” and “R6 rep 2” are homologous and are both type 4, shown as blue). Repeat elements that contain the *MDox* insertion were concatenated (i.e., the *MDox* insertion was excised) and are denoted with an asterisk.

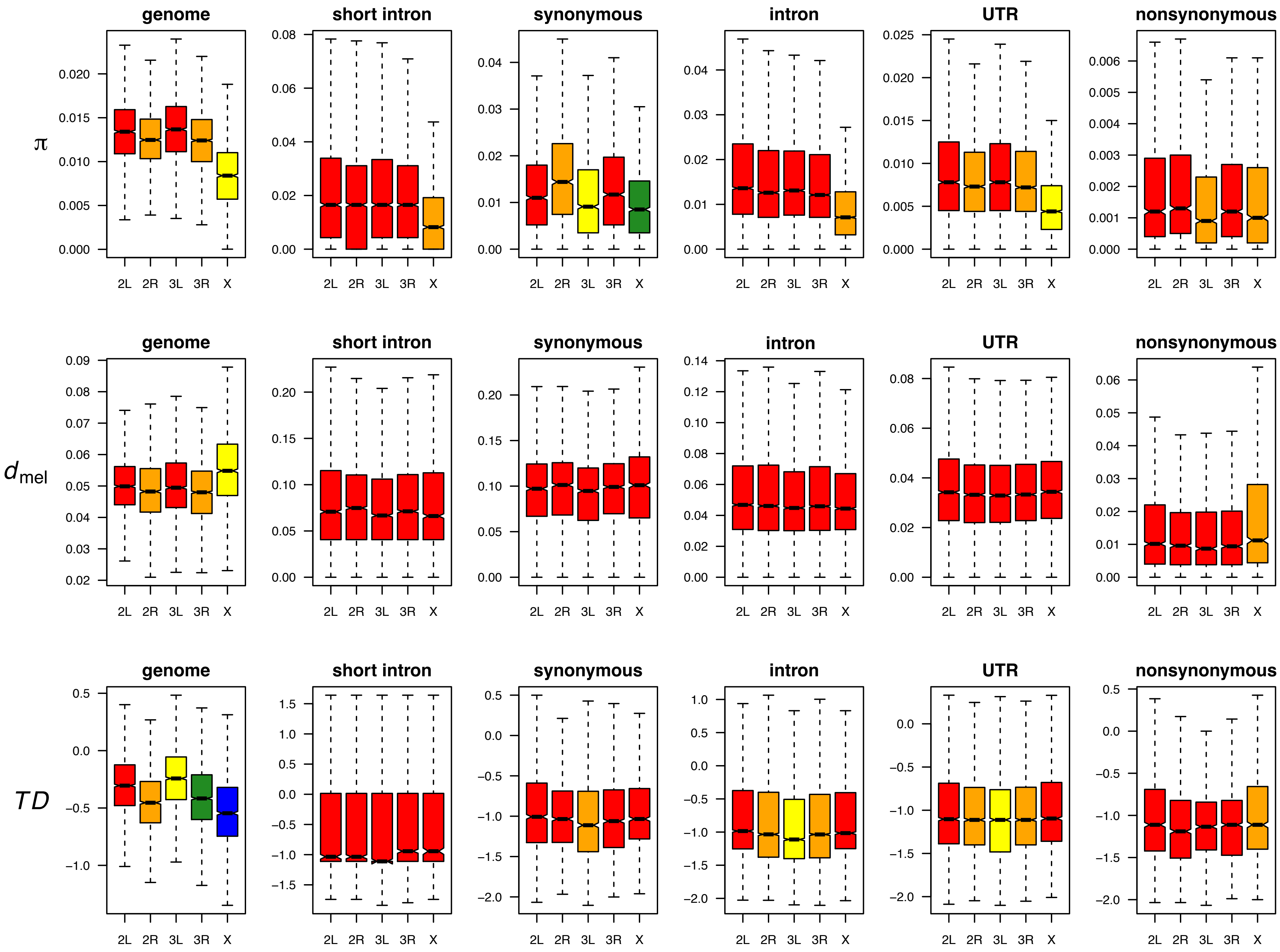

π
d_{mel}
TD
genome
short intron
synonymous
intron
UTR
nonsynonymous
2L 2R 3L 3R X

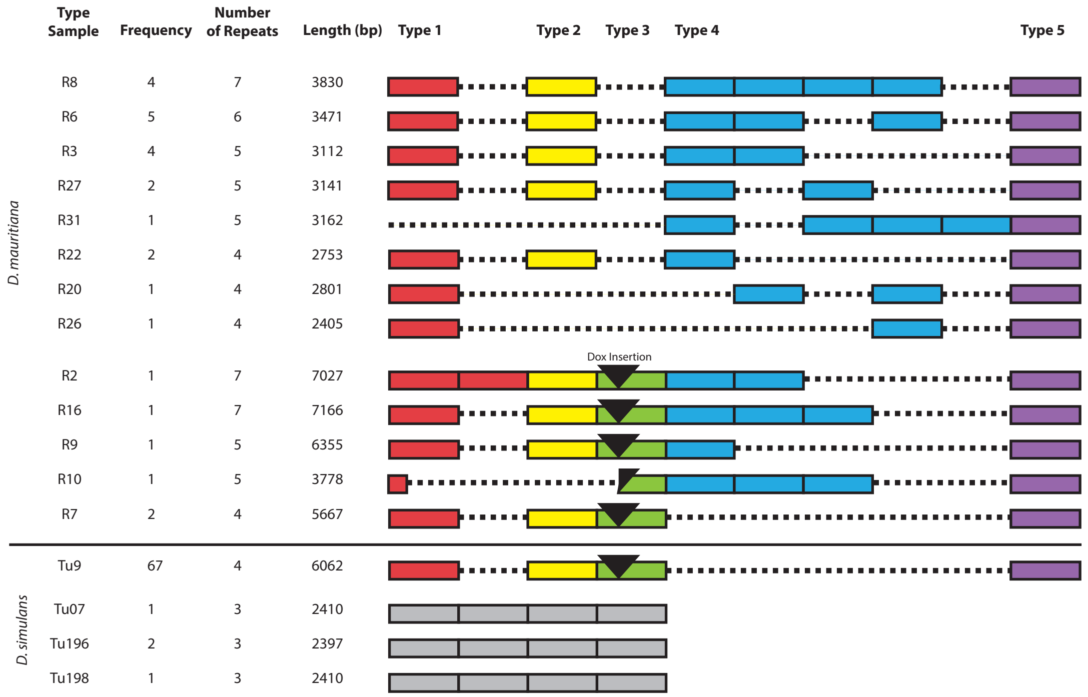

Type Sample
Frequency
Number of Repeats
Length (bp)
Type 1
Type 2
Type 3
Type 4
Type 5
D. mauritiana
R8 4 7 3830
R6 5 6 3471
R3 4 5 3112
R27 2 5 3141
R31 1 5 3162
R22 2 4 2753
R20 1 4 2801
R26 1 4 2405
Dox Insertion
R2 1 7 7027
R16 1 7 7166
R9 1 5 6355
R10 1 5 3778
R7 2 4 5667
D. simulans
Tu9 67 4 6062
Tu07 1 3 2410
Tu196 2 3 2397
Tu198 1 3 2410

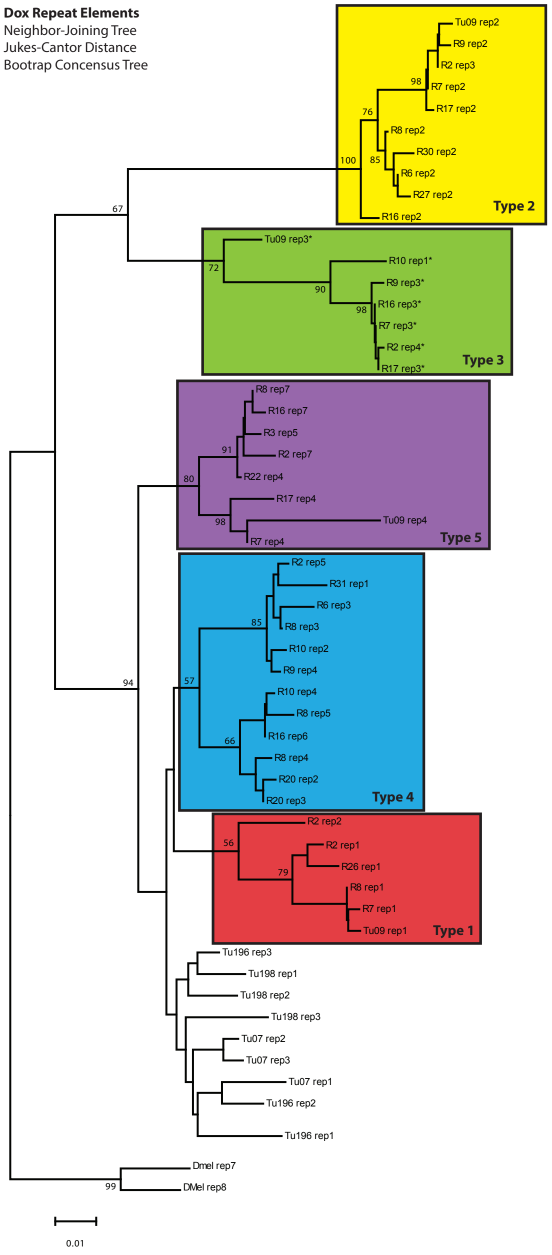

Dox Repeat Elements
Neighbor-Joining Tree
Jukes-Cantor Distance
Bootrap Concensus Tree
Tu09 rep2
R9 rep2
R2 rep3
R7 rep2
R17 rep2
R8 rep2
R30 rep2
R6 rep2
R27 rep2
R16 rep2
Type 2
Tu09 rep3*
R10 rep1*
R9 rep3*
R16 rep3*
R7 rep3*
R2 rep4*
R17 rep3*
Type 3
R8 rep7
R16 rep7
R3 rep5
R2 rep7
R22 rep4
R17 rep4
Tu09 rep4
R7 rep4
Type 5
R2 rep5
R31 rep1
R6 rep3
R8 rep3
R10 rep2
R9 rep4
R10 rep4
R8 rep5
R16 rep6
R8 rep4
R20 rep2
R20 rep3
Type 4
R2 rep2
R2 rep1
R26 rep1
R8 rep1
R7 rep1
Tu09 rep1
Type 1
Tu196 rep3
Tu198 rep1
Tu198 rep2
Tu198 rep3
Tu07 rep2
Tu07 rep3
Tu07 rep1
Tu196 rep2
Tu196 rep1
Dmel rep7
DMel rep8
98
76
100
85
67
72
90
98
91
80
98
85
94
57
66
56
79
99
0.01

	Type Sample	Frequency	Number of Repeats	Length (bp)
D. mauritiana	R18	1	4	5260
	R20	1	4	5152
	R6	23	2	4544
	R19	1	1	4179
D. simulans	Tu9	65	2	4508
	Tu07	1	3	3618
	Tu184	1	3	3604
	Tu196	1	2	3349
	Tu197	1	1	2845

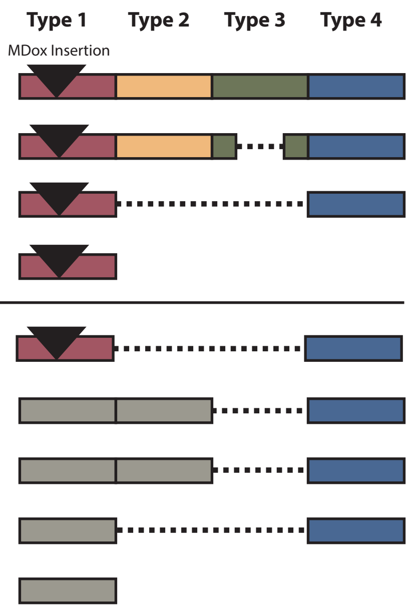

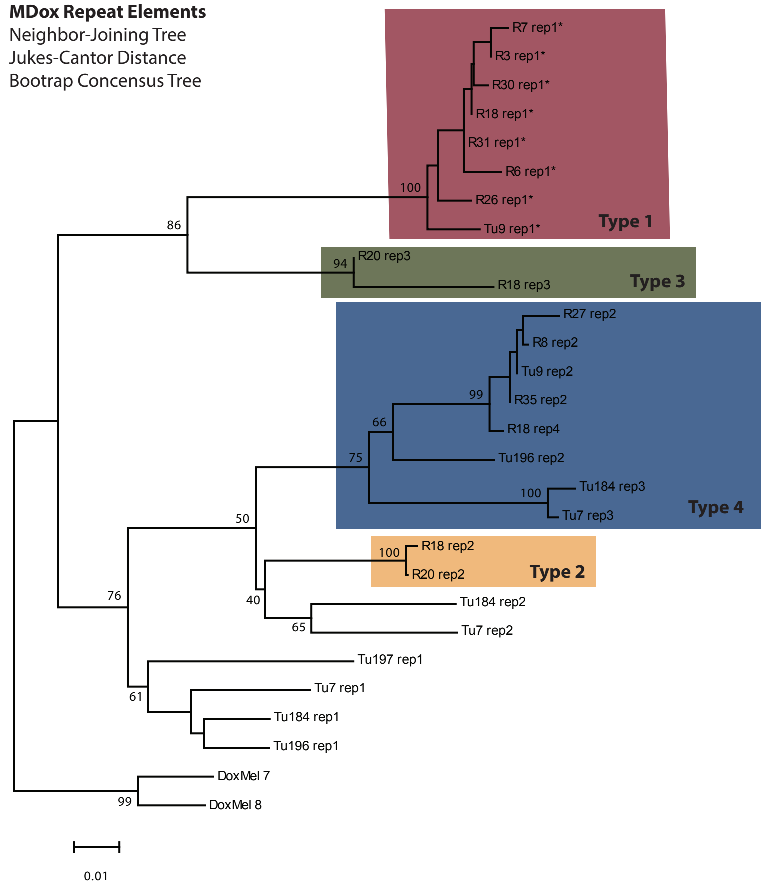

Supplement: Supplementary Data [file supp_evu198_Supplementary_Material.pdf]
